# Hemorrhagic shock secondary to button battery ingestion

**DOI:** 10.1590/1516-3180.2014.1323697

**Published:** 2014-04-28

**Authors:** Naomi Andreia Takesaki, Marcelo Conrado dos Reis, Maria Luisa Ferreira de Miranda, Emílio Carlos Elias Baracat

**Affiliations:** I MD. Attending Physician, Department of Pediatrics, School of Medical Sciences, Universidade Estadual de Campinas (Unicamp), Campinas, São Paulo, Brazil; II MD, Attending Physician and Head of Pediatric Emergency Service, Hospital de Clínicas, Universidade Estadual de Campinas (Unicamp), Campinas, São Paulo, Brazil; III MD, Attending Physician and Head of Pediatric Ward, Hospital Estadual Sumaré, Sumaré, São Paulo, Brazil; IV MD, PhD. Associate Professor, Department of Pediatrics, School of Medical Sciences, Universidade Estadual de Campinas (Unicamp), Campinas, São Paulo, Brazil

**Keywords:** Foreign bodies, Electric power supplies, Gastrointestinal hemorrhage, Shock, Child, Corpos estranhos, Fontes de energia elétrica, Hemorragia gastrointestinal, Choque, Criança

## Abstract

**CONTEXT:**

Button battery ingestion is a frequent pediatric complaint. The serious complications resulting from accidental ingestion have increased significantly over the last two decades due to easy access to gadgets and electronic toys. Over recent years, the increasing use of lithium batteries of diameter 20 mm has brought new challenges, because these are more detrimental to the mucosa, compared with other types, with high morbidity and mortality. The clinical complaints, which are often nonspecific, may lead to delayed diagnosis, thereby increasing the risk of severe complications.

**CASE REPORT:**

A five-year-old boy who had been complaining of abdominal pain for ten days, was brought to the emergency service with a clinical condition of hematemesis that started two hours earlier. On admission, he presented pallor, tachycardia and hypotension. A plain abdominal x-ray produced an image suggestive of a button battery. Digestive endoscopy showed a deep ulcerated lesion in the esophagus without active bleeding. After this procedure, the patient presented profuse hematemesis and severe hypotension, followed by cardiorespiratory arrest, which was reversed. He then underwent emergency exploratory laparotomy and presented a new episode of cardiorespiratory arrest, which he did not survive. The battery was removed through rectal exploration.

**CONCLUSION:**

This case describes a fatal evolution of button battery ingestion with late diagnosis and severe associated injury of the digestive mucosa. A high level of clinical suspicion is essential for preventing this evolution. Preventive strategies are required, as well as health education, with warnings to parents, caregivers and healthcare professionals.

## Abstract (pt)

**CONTEXTO:**

A ingestão de bateria em disco é queixa frequente em pediatria. As complicações graves decorrentes de ingestão acidental têm aumentado significativamente nas últimas duas décadas, devido ao fácil acesso aos aparelhos e brinquedos eletrônicos. Nos últimos anos, o aumento do uso de baterias de lítio com diâmetro de 20 mm trouxe novos desafios, por serem mais prejudiciais para a mucosa em comparação com outros tipos, com elevada morbidade e mortalidade. As queixas clínicas, muitas vezes inespecíficas, podem levar ao atraso no diagnóstico, aumentando o risco de complicação grave.

**RELATO DE CASO:**

Menino de cinco anos, com queixa de dor abdominal há 10 dias, é trazido ao serviço de emergência com quadro clínico de hematêmese há duas horas. Na admissão, apresentava palidez, taquicardia e hipotensão. Imagem sugestiva de bateria em disco foi visualizada na radiografia simples de abdome. A endoscopia digestiva demonstrou lesão ulcerada profunda no esôfago sem sangramento ativo. Após o procedimento, o paciente apresentou hematêmese profusa e hipotensão grave, seguidos de parada cardiorrespiratória (PCR), revertida. Submetido a laparotomia exploradora de urgência, apresentou novo episódio de PCR, sem reversão. A bateria foi removida por exploração retal.

**CONCLUSÃO:**

Este caso descreve a evolução fatal de ingestão da bateria em disco com diagnóstico tardio e associação com lesão grave de mucosa digestiva. Alto nível de suspeita clínica é obrigatório para evitar tal evolução. As estratégias de prevenção são necessárias, bem como a educação em saúde, com alerta aos pais, cuidadores e profissionais de saúde.

## INTRODUCTION

Accidents during childhood are an important cause of morbidity and mortality among children, and are the primary cause of death between the ages of 1 and 19 years.^1^ ^,^ ^2^ Ingestion/aspiration of foreign bodies has been identified as one of the top five types of accidents occurring in the pediatric age group, with peak incidence at between one and three years of age.^1^ ^-^ ^3^ Button battery ingestion represents about 2% of foreign body ingestion cases.^4^ ^,^ ^5^ Data from the American Poison Control Center has shown that the estimated incidence of button battery ingestion in the US is 1/11.1 million inhabitants, consisting mostly of accidental intake.^6^ Although the majority of battery ingestion is benign, one in every 1000 cases results in harm, especially when there is esophageal impaction.^5^ ^,^ ^7^

Most patients are asymptomatic, and the signs and symptoms are related to the anatomical location of the battery, length of time lodged at this location, size, composition and quantity ingested. In symptomatic cases, coughing, dysphagia, dyspnea, nausea, abdominal pain and vomiting may occur, in addition to nonspecific signs such as fever, irritability and food refusal.^4^ ^,^ ^5^ ^,^ ^8^

Imaging tests may help identify the foreign body and should be carefully analyzed, looking for evidence that suggests the presence of batteries, such as the double-contour sign in the anteroposterior projection of simple x-rays and the "step" sign in lateral view.^9^ ^,^ ^10^

Esophageal injury relating to battery ingestion is caused basically by three mechanisms: local electric power generation with burning of the mucosa, mechanical pressure necrosis and alkaline battery content leakage. Although rare, it is possible for poisoning due to absorption of mercury to occur, although this cannot occur in batteries containing lithium, manganese or other metals.^4^ ^,^ ^5^ ^,^ ^11^ The complications relating to ingestion of button batteries include esophageal perforation, perforation of Meckel's diverticulum, esophageal stenosis, tracheoesophageal fistula, paralysis of vocal cords, spondylodiscitis, mediastinitis, bleeding and death.^9^ ^,^ ^11^ ^-^ ^20^

The therapeutic approach in cases of batteries in the esophagus is to undertake immediate withdrawal by means of endoscopy. For batteries in the stomach, there is still some controversy in the literature regarding case management, but this is mostly guided by the patient's symptoms.^21^ After battery withdrawal by means of endoscopy, treatment with anti-reflux drugs, antibiotics or corticosteroids may be administered. Use of esophageal stents is still controversial, with low levels of improvement shown.^22^

Failure to recognize esophageal foreign bodies or inappropriate management of such cases may lead to life-threatening conditions. In this context, we describe the case of a preschool patient with fatal evolution after accidental ingestion of a button battery.

## CASE REPORT

**Figure 1:**
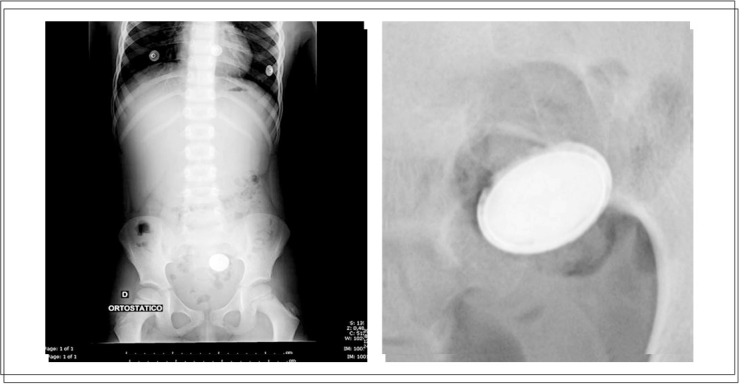
Simple abdominal radiography showed a radiopaque image with double circular halo outline in the distal colon.

A five-year-old male patient who had been complaining of abdominal pain for the past 10 days was taken to the emergency referral unit of Sumaré State Hospital with a condition of hematemesis (two episodes in the last two hours). No other symptom or important antecedent was reported by the family. The mother said that she had sought medical services twice during the period, and the child had been evaluated and released since there were no symptoms. At admission he presented pallor, tachycardia (150 beats per minute, bpm) and hypotension (60 mmHg), and volume expansion with saline solution was started. The blood tests upon admission showed: hemoglobin (Hb): 8.9 g%; hematocrit: 27%; platelets: 161,000; International Normalized Ratio (INR): 1.09; and Ratio (R): 0.87. Simple abdominal radiography showed a radiopaque image with a double circular halo outline in the distal colon suggestive of a button battery (the mother was unaware of this ingestion) (Figure 1).

Within two hours, he presented two more episodes of hematemesis, with Hb falling to 6.8 g%. A transfusion of packed red blood cells (10 ml/kg) was administered. Upper gastrointestinal endoscopy was performed in the surgical room (four hours after arrival), which showed a deep ulcerated lesion in the distal third of the esophagus, occupying 70% of the circumference and 5 cm in length. The bottom of the ulcer was covered by fibrin and a large clot had adhered to it, without active bleeding. After withdrawal of the endoscope, he presented new profuse hematemesis, as well as severe hypotension, and a Sengstaken-Blakemore tube was introduced. The case evolved with major abdominal distension and cardiopulmonary arrest, which was reversed by means of chest compressions and adrenaline.

Because of the gradually increasing abdominal distension, it was decided that exploratory laparotomy should be performed by the pediatric surgeons. Through this, large amount of blood was found in the stomach, without any perforation of the digestive tract, and no blood was found in the peritoneal cavity. During the procedure, the child presented a new cardiopulmonary arrest, but did not recover from it. The button battery was removed by means of rectal manipulation during the procedure.

## DISCUSSION

Nowadays, button batteries are part of the technological reality experienced by children. The data show that there has been exponential growth in the frequency and severity of injuries caused by ingestion of these products. Between 1990 and 2009, there were more than 65,000 accidents with batteries among individuals under the age of 18 years in the United States, with an increase from 4 to 7.4 cases/100,000 children over that period.^23^ Ingestion accounted for the largest contingent, with 76% of the accidents, and also accounted for an increase of 80% in the number of cases over the past 8 years.^24^ As a result of changes to the types of batteries swallowed, severe complications and deaths due to battery ingestion have increased by around sevenfold, mostly among children younger than 4 years of age.^8^ ^,^ ^25^ ^,^ ^26^

A review of the literature was conducted through an online search for the MeSH terms Electric Power Supplies, Foreign Bodies, Gastrointestinal Hemorrhage and Shock, Hemorrhagic, in Medline (via PubMed); the EMTREE terms Electric Battery, Gastrointestinal Hemorrhage and Hemorrhagic Shock, in Embase (via Elsevier); and the MeSH/DeCS terms Foreign Bodies, Gastrointestinal Hemorrhage and Shock, Hemorrhagic, in Lilacs (via Bireme) (Table 1).

**Table 1:** Database search results for foreign body, electric battery, gastrointestinal hemorrhage and hemorrhagic shock on May 22, 2013

Electronic databases	Search strategies	Results
Medline (PubMed)	(Electric Power Supplies) AND (Foreign Bodies) AND (Gastrointestinal Hemorrhage) AND (Shock, Hemorrhagic)	No original articles, case reports or review articles
Embase (Elsevier)	(Electric Battery) AND (Gastrointestinal Hemorrhage) AND (Hemorrhagic Shock)	No original articles, case reports or review articles
Lilacs (Bireme)	((Foreign Bodies) OR (Cuerpos Extraños) OR (Corpos Estranhos)) AND ((Gastrointestinal Hemorrhage) OR (Hemorragia Gastrointestinal) OR (Hemorragia Gastrointestinal)) AND ((Shock, Hemorrhagic) OR (Choque Hemorrágico) OR (Choque Hemorrágico))	No original articles, case reports or review articles

The first report of death resulting from ingestion of button batteries was in 1977, which occurred in the case of an infant who ingested a photographic camera battery.^27^ Since then, numerous reports have been published in the medical literature, with 13 fatal cases identified in a recent review study.^28^ Exsanguination was shown to be the cause of death in 10 cases, seven of which occurred after 2004, mostly secondary to tracheoesophageal fistula. All cases of fatal bleeding occurred in the age group from 11 months to 3 years of age, and the average duration of esophageal impaction was from 10 hours to 14 days. In six cases, the battery was removed before bleeding could occur. Evidence of bleeding, such as hematemesis or melena, was present in 70% of the fatal cases in the days or hours prior to the hemorrhage that led to death, which makes it important to search for signs in the clinical histories of stable patients.^25^ ^,^ ^28^

The increasing severity of injuries associated with ingestion of batteries over the last two decades has taken place in parallel with the manufacturers' transition to production of lithium batteries. Because lithium is a light metal, lithium batteries have higher efficiency, high electrochemical energy-density and longer life, thus making them commonly used in domestic appliances. However, their high voltage (3 V) makes their ingestion more dangerous than conventional batteries (1.5 V). Esophageal lesions have been observed within the first hour of impaction in the esophagus, in experimental studies on dogs.^29^ In humans, esophageal burns have been described within less than two and a half hours after ingestion, and perforations may occur about five hours after the accident.^5^ ^,^ ^17^ ^,^ ^25^ In addition, the greater diameter of most lithium batteries (20 mm) makes them more susceptible to lodging in the esophagus of children. There was an increase in ingestion of batteries of diameters between 20 and 25 mm, from 1% to 18% between 1990 and 2008, in parallel with increased ingestion of lithium batteries (from 1.3% to 24%) over the same period. About 92% of the batteries identified in cases of greater severity and lethality were lithium batteries of diameter 20 mm.^25^

Three basic injury mechanisms have been described in cases of battery contact with the mucosa of the gastrointestinal tract: generation of electric current with local burning; mechanical pressure causing necrosis; and leakage of alkaline content. In lithium batteries, their high voltage results in higher electric current generation capacity, with greater amounts of tissue fluid hydrolysis and production of hydroxides higher than in other battery types, which increases the potential for injury. Severe burns are observed in the area adjacent to the negative pole of the impacted battery, since electric current originates at this pole. Thus, battery position in the esophagus works as a predictor of severity.^25^

Most accidents that have evolved to complications or death present the common factor that the diagnosis was delayed. This has often been due to the nonspecific presentation, especially in unwitnessed cases, like in the present case. In acute witnessed cases, the battery needs to be removed within two hours in order to avoid injury to the esophagus. This shorter window of opportunity, in comparison with previous descriptions, is probably related to the introduction of new types of batteries.

A simple x-ray examination is the preferred method in cases of suspicion of battery ingestion, whether the patients are symptomatic or not.^5^ The image must be evaluated properly, looking for the double outline stepped sign in anteroposterior and lateral views. Cases of unknown ingestion with radiological images similar to coins should be assumed to be batteries and conducted as such in order to prevent complications and death.

All studies have emphasized that immediate withdrawal by means of endoscopy should be undertaken if the battery is lodged in the esophagus, with direct viewing of the mucosa. In cases in which the battery has already reached the stomach and the patient is asymptomatic, divergences exist with regard to case management. Litovitz et al. suggested that the x-ray examination should be repeated four days later (or sooner if symptoms appear) and, if the battery is still in the stomach, endoscopic withdrawal should then be indicated.^25^ If, after the withdrawal, lesions are observed in the mucosa, complementary examinations (contrasted x-ray examinations, bronchoscopy or computed tomography) must be performed to evaluate possible complications such as fistula or perforations. In addition, in serious cases, vital signs need to be monitored in a hospital environment even after withdrawal of the battery, given that there have been cases of exsanguination and death occurring 18 days after the endoscopy.^25^

## CONCLUSION

This case of fatal evolution relating to battery ingestion is presented with the intention of informing pediatricians of the dangers relating to this type of accident. Healthcare professionals need to maintain a high level of suspicion, given that the delayed and sometimes few symptoms can frequently be mistaken for childhood diseases that occur more commonly.

Pediatric emergency services must be prepared to act quickly and promptly in cases of complications caused by battery ingestion. Specific guidance for parents and caregivers are also required, and constitute important preventive measures for this type of injury.
